# Understanding forgotten exposures towards achieving Sustainable Development Goal 3: a cross‐sectional study on herbal medicine use during pregnancy or delivery in Tanzania

**DOI:** 10.1186/s12884-021-03741-5

**Published:** 2021-04-01

**Authors:** Anna Tengia-Kessy, George Chombe Msalale

**Affiliations:** 1 Department of Community Health, School of Public Health and Social Sciences, Muhimbili University of Health and Allied Sciences, P. O. Box 65015, Dar es Salaam, Tanzania; 2 Kitete Regional Referral Hospital, P. O. Box 22, Tabora, Tanzania

**Keywords:** Herbal medicine, Pregnancy, Sustainable Development Goal 3, Tanzania

## Abstract

**Background:**

In most of the sub-Sahara African countries, use of herbal medications is widely practiced during pregnancy or delivery for various reasons despite uncertainties on their pharmacological profiles. Use of unregistered herbal medicines has the potential of causing adverse health effects to the mother and the newborn, thus deterring achievement of Sustainable Development Goal 3, which aims to “ensure healthy lives and promote well-being for all at all ages”. One of the targets is on reduction of morbidity and mortality among mothers and newborns. This study investigated use of herbal medicines and predictors of usage during pregnancy or delivery as a forgotten exposure towards understanding some of the challenges in achieving Sustainable Development Goal 3.

**Methods:**

This cross-sectional quantitative study gathered information from women who delivered a live-born baby in the preceding two years. Using a two-stage-sampling technique, women attending reproductive, maternal and child health clinics in Tabora were selected and interviewed. Proportions were compared using chi-square test and Poisson regression analysis was performed to determine independent correlates of herbal medicine use.

**Results:**

Of 340 recruited women, 208 [61.2 %; 95 % confidence interval: 55.4, 66.3 %] used herbal medicines during pregnancy or delivery. Major reasons for use included accelerating labour, 81 (38.9 %) and reducing labour pains, 58 (27.9 %). Women who made less than four antenatal visits had a 24 % higher adjusted prevalence ratio of using herbal medicines as compared to those who had at least four visits [adjusted prevalence ratio:1.24; 95 % confidence interval: 1.02, 1.50, p = 0.03]. Furthermore, the adjusted prevalence ratio of using herbal medicines was 35 % higher among women who were not discouraged by health care providers against their use as compared to those who were discouraged (adjusted prevalence ratio: 1.35; 95 % confidence interval: 1.13, 1.60, p = 0.01).

**Conclusions:**

Use of herbal medicines during pregnancy or delivery among women in Tanzania is common. Independent predictors of herbal medicine use were number of antenatal visits and stance of maternity health care providers on their use. Comprehensive investigations on the magnitude, patterns and predictors of use of herbal medicines during pregnancy or delivery are warranted.

**Supplementary Information:**

The online version contains supplementary material available at 10.1186/s12884-021-03741-5.

## Background

Recent global estimates indicate that pregnancy and childbirth-related complications claimed about 300,000 lives of women annually and were observed mostly in sub-Sahara African countries [1]. Tanzania, like many other sub-Sahara African countries, has unacceptably high rates of perinatal mortality, 39 deaths per 1000 pregnancies; neonatal mortality, 25 deaths per 1000 live births; and maternal mortality ratio of 556 per 100,000 live births [2]. Most of these deaths can be prevented based on the adequacy of clinical management and quality of care that are provided during pregnancy, delivery and postpartum periods [3].

The world has unanimously adopted the Sustainable Development Goals (SDGs) as a joint response to the prevailing under development and high levels of human suffering [1]. Among them, SDG 3 is to “ensure healthy lives and promote well-being for all at all ages”. This includes reduction of morbidity and mortality among women and newborns. Since maternal, newborn and child deaths are caused by factors attributable to pregnancy, childbirth and poor quality of health services, achieving the SDG 3 planned target in the reduction of maternal mortality will also bring to an end most of the preventable deaths of children less than five years of age.

Despite the United Nation’s SDGs and consequent national policy adaptation, there remains a dearth of comprehensive information on possible factors that could deter achieving SDG 3 by 2030. Obtaining such information will facilitate evaluation of best practices that will inform key decisions. Such decisions will complement the ongoing efforts to promote maternal health by increasing facility-based deliveries from the current 60–90 % by 2030. This is in the perspective that more women will attend antenatal care services and deliver in an environment that is conducive for supervised and hence safe births. At the same time, use of health facilities will minimize exposures to unsafe traditional systems, including use of herbal medicines (HMs) during pregnancy or delivery.

Considerable efforts by the sub-Saharan countries towards attaining SDG 3 are focused on improving skilled attendance at delivery as an end, with insufficient attention to some of the practices that may contribute to the observed maternal and newborn burden of disease. Increasing diversification in the causes of maternal mortality and morbidity underscores the importance of assessing and addressing not only the proximal causes of maternal death, but also the broad range of distal social determinants of health, including the use of HMs [4]. In countries like Tanzania where the maternal mortality ratio remains high, antenatal education is a priority so as to increase birth preparedness and readiness for complications. This education focuses on birth plans such as the birthplace, birth attendant and transportation; health facility for complications; and related expenses. Exposures, such as use of HMs are not a routine part of the information package provided. Thus, the antenatal health care providers only discuss use of HMs at their own discretion. This study takes the first step towards contributing to understanding these exposures; starting with the use of HMs in pregnancy or delivery that may also be common in many other countries.

Existing evidence supports high magnitude of exposure to HMs whose safety profiles remain unknown [5], subjecting women and newborns to potentially toxic substances before, during or after birth. For example, a systematic review of literature [6] focusing on use of HMs to induce labour by pregnant women revealed proportions of HMs use in the most recent pregnancy ranging from 6.5 % in Ghana to 80 % in Uganda [7, 8]. Similarly, high proportions (23 %) of women in Tanzania use HMs during pregnancy and for labour induction. Some of the commonly used HMs in Tanzania, Ethiopia and Middle East include a wide variety of herbs such as ginger (*Zingiber officinale*), onions (*Allium cepa*) and Neem *(Azadirachta indica*) [9–11].

Use of HMs among pregnant women may depend upon social status, ethnicity and cultural tradition, and the indications for the use may vary across regions and can be mother or child- related [12]. Some of the provided reasons for using HMs during pregnancy or delivery include prevention of incidents of nausea and vomiting, improving abdominal muscle tone and building stamina during labour and delivery (strengthening pregnancy); health of the woman and the fetus; ensuring positive pregnancy outcomes and easing labour [9, 13–16]. The conviction that HMs have the ability of inducing and accelerating labour; alleviating labour pains, enhancing removal of a retained placenta, as well as toning the uterine muscles post-delivery [13] are additional reasons for their usage. Moreover, some women use HMs as remedies to protect the fetus from evil spirits in-utero and to have a healthy baby [14, 17]. Timing of intense exposures to the possible toxic materials coincides with very early stages of labour, posing risk to both mother and newborn [9]. Use of HMs at this phase is aimed to stimulate uterine muscles resulting to stronger contractions and thus, hastening labour [8]. The HMs may be taken via the oral route in which they are most often consumed as a strong tea or chewed; rectal or vaginal routes and sometimes rubbing them on the pregnant abdomen [18].

Arguments on effectiveness of HMs are, however, subjective and unjustified given the fact that most of the HMs used across sub-Saharan Africa are associated with important research gaps as several of them have never been botanically identified [5]. Therefore, their use during pregnancy or delivery is a subject of concern since some plants might have parts that contain natural toxins that could be dangerous if they cross the placental barrier. Luckily, modern health care system has readily available and safe interventions such as the use of oxytocin and artificial rupture of membranes to facilitate timely and safe deliveries.

With the pressing need to attain SDG 3 by the year 2030, exposure to HMs during pregnancy or delivery must be restricted in order to improve maternal and newborn health. Therefore, studying usage of HMs in relation to maternal health is a public health priority in achieving SDG 3 in many countries, Tanzania inclusive. Thus, the objective of this study was to investigate use of HMs and predictors of usage during pregnancy or delivery as an input towards efforts for achieving the goal of improving maternal and newborn health.

## Methods

### Study design

A quantitative descriptive cross-sectional study was carried out among 340 mothers attending reproductive, maternal and child health (RMCH) services in Tabora region.

### Study setting

The study was conducted in Tabora municipal, one of the seven districts of Tabora region. The region is among the 31 administrative regions of Tanzania, and is located in the central-western part of Tanzania mainland. In the context of administrative structures in mainland Tanzania, a municipal is an urban authority responsible for the administration and development of the urban area. Urban authorities consist of city councils (e.g., Dar es Salaam); municipal councils (e.g., Tabora); and town councils, whereas included in the rural authorities are the district councils with township and village council authorities.

Administratively, Tabora municipality has two divisions; namely Tabora North and South with 14 and 15 wards (administrative units) respectively. The municipality has 44 health facilities of which 36 are public. These include three hospitals, three health centers and 30 dispensaries. Of these public health facilities, 34 provide RMCH services. The most recent projected population of the municipality based on the 2012 national population census is 262,747 of whom women of childbearing age (15‒49 years) constitute about 10 % [19].

### Study participants

The study participants comprised of women who delivered a live-born baby between September 2016 and September 2018 and attending RMCH services in Tabora municipal health facilities. The eligibility criterion is that such women must have lived in Tabora municipality during the pregnancy or delivery of the index child. Tabora North and South divisions have 18 and 16 health facilities respectively, providing RMCH services.

### Sample size estimation and sampling process

The sample size was calculated using the formula for estimating a single proportion, Z^2^p(1-p)/m^2^; whereby Z is the critical value of the normal distribution at 5 % level of significance, p is an estimated proportion (23 %) of pregnant women using HMs [9]; and 5 % for m, an estimated margin of error. Allowing for a non-response rate of 20 %, the ultimate sample size was 342 women attending RMCH clinics.

Proportionately, we estimated nine and eight health facilities from Tabora North and South divisions, respectively. The first stage involved selecting health facilities from each of the two divisions using systematic sampling technique. In the second stage, we randomly selected women who met the study criterion from the facilities. The number of women selected in each facility was based on the proportion a facility contributed to maternal attendances in all selected facilities in a division put together in the previous month. All consenting women were interviewed in December 2018.

### Study variables and measurements

The dependent variable was HM use during the most recent pregnancy or delivery, with response categories of Yes or No. The independent variables comprised of socio-demographic characteristics of interest including (1) *age* (in completed years); (2) *current marital status* (never in union, currently in union, previously in union); (3) *highest education level attained* (none/primary incomplete, primary complete, secondary and above); and (4) *occupation of respondent/spouse* (housewife/peasant, self-employed, formerly employed). Others included (5) *distance to the nearest health facility*; (6) *number of antenatal clinic visits*; (7) *perceived availability of HMs* (easily available, not easily available); (8) *perceived safety of HMs* (safe, not safe); and (9) *stance of maternity health care provider on the use of HMs* (did not discourage use, discouraged use). Quantitative independent variables were further categorized as: age of the mother (16 to 25; 26 to 35, 36 + years); distance to the nearest health facility (> 5 km, ≤ than 5 km); and number of antenatal visits during last pregnancy (less than 4, 4 or more visits).

### Study procedure and data collection

A pre-coded and pre-tested questionnaire was used to collect information on individual’s demographic characteristics, availability and accessibility of HMs; use of HMs during the most recent pregnancy or delivery and the reasons for use. Also, distance to the nearest public health facility, awareness of any adverse effects of using HMs for obstetrical purposes and whether or not the health care providers discouraged use of HMs during antenatal clinic (ANC) attendance were inquired.

The questionnaire was initially developed in English and later translated into Kiswahili which is the national medium of communication. It was then back translated into English. When consistency was achieved, the Kiswahili version was adopted for the interviews. We recruited a team of four registered nurses as interviewers. These nurses were assigned to collect data from facilities other than their working sites. They were initially informed on the purpose and ethical aspects of the study and then trained on interviewing techniques. The research team administered the questionnaire to women in a selected private environment within the health facility on exit.

We assumed that all selected women were most likely to remember use of HMs during their most recent pregnancy or delivery, thus, in position to give a self-report. For content validity of the tool and other methodological issues as described by Castillo-Montoya [20], we ensured interview questions aligned with the research objectives; and discussed the protocol with an obstetrician. We used the feedback to improve the interview questions. To improve the face validity, we pre-tested the instrument among 10 % of the estimated sample size of women in a nearby health facility that was not earmarked for the main study. The questionnaire was modified as necessary based on the pre-test findings. As part of quality assurance, the process of data collection was closely supervised to address any problems encountered. Filled-in questionnaires were checked for completeness of the data and rectifications made as appropriate.

### Sources of bias and mitigations

Reporting bias, which could have affected the findings in this study, was minimized by the use of well-trained registered nurses in data collection. These nurses were RMCH services providers in clinics other than the assigned site of data collection to curtail socially desirable responses from the women. Recall bias, another potential source of prejudice was minimized by recruiting only women who gave birth within the preceding two years. Such women were more likely to remember use of HMs compared to women whose last pregnancy or delivery was three years or longer.

### Statistical analysis

Before the actual data analysis, the quantitative data were coded and entered into a computerized data-base using Statistical Package for Social Sciences, Version 24; and checked for completeness and consistency. Initially, by running frequencies of all variables, we were able to detect possible out-of-range values that were corrected. Prevalence was calculated as proportion of study participants who used HMs whereas the denominator was all women enrolled in the study. In the univariate analysis, categorical variables were summarized as proportions. We then performed bivariate analysis and used Pearson’s Chi-square test to assess the association of selected independent variables with use of HMs. To assess independent predictors of use of HMs, factors with p-value < 0.2 in the binary analyses were selected to enter into a modified Poisson regression model with robust standard error estimation [21]. Factors that were loaded into the model included occupation, frequency of ANC visits, distance to the nearest facility, perceived safety of HMs and the stance of health care providers regarding use of HMs in pregnancy or delivery. Effect sizes of the different factors on use of HMs are presented as Adjusted Prevalence Ratios (aPR) and their corresponding 95 % confidence intervals (CI); and the significance level was set at 5 %.

## Results

**Fig. 1:**
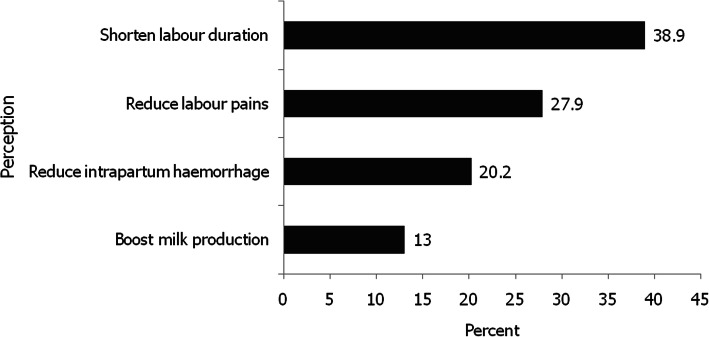
Most perceived benefits of using herbal medicines during pregnancy or delivery among women in Tabora, Tanzania

A total of 340 women were recruited (recruitment rate of 99.4 %) and participated in this study. Two women (aged 28 and 37 years) declined to be interviewed for undisclosed reasons. A large proportion of women, 208 (61.2 %; 95 %CI: 55.4, 66.3 %) used HMs during their most recent pregnancy or delivery. While the majority, 138 (66.3 %), used HMs for obstetrical reasons at least on three different occasions, 16 (7.7 %) and 54 (26.0 %) used HMs on either one or two occasions respectively. Of the 208 women who used HMs, 57 (27.4 %) viewed them as easily available and 83 (39.9 %) considered them safe. The key modes of administration included oral 135 (64.9 %) and intra-vaginal 59 (28.4 %) routes. The main perceived benefits of using HMs among the 208 women who used them included shortening of labour duration, 81 (38.9 %) and alleviating labour pains, 58 (27.9 %), as displayed in Fig. 1.

Less than half, 164 (48.2 %) of the respondents were aware of adverse effects of using HMs in pregnancy or delivery. Significantly more women, 93.3 % (153/164), who were aware of the adverse effects of using HMs used them as compared to 31.3 % (55/176) who were not aware (*p* < 0.01). Irrespective of HM use status, uterine rupture 70 (20.6 %), excessive vaginal bleeding 51 (15.0 %), and possibility of death of the mother and/or the baby 21 (6.2 %) were stated as untoward consequences of using HMs.

In the bivariate analyses (Table 1), herbal medicine use during pregnancy or delivery was significantly associated with distance to the nearest health facility (*p* = 0.021), number of antenatal visits (*p* = 0.011), perceived safety of HMs (*p* < 0.01) and the stance of health care providers (*p* < 0.01).

**Table 1:** Bivariate analysis of selected characteristics with herbal medicine use during pregnancy or delivery in Tabora, Tanzania

Characteristic	Total	Herbal use Number (%)	Chi-square, ***p***-value*
**Age group (years)**			1.62, 0.431
16-25	83	46 (55.4)	
26-35	142	88 (62.0)	
36+	115	74 (64.3)	
**Marital status**			0.67, 0.717
Never in union	25	14 (56.0)	
Currently in union	285	174 (61.1)	
Previously in union	30	20 (66.7)	
**Education level**			2.86, 0.239
None/prim incomplete	87	50 (57.5)	
Primary complete	175	104 (59.4)	
Secondary and above	78	54 (69.2)	
**Occupation of mother**			3.63, 0.163
Peasant/housewife	177	108 (61.0)	
Self-employed	127	73 (57.5)	
Formerly employed	36	27 (75.0)	
**Occupation of spouse**^**a**^			4.59, 0.101
Peasant	159	103 (64.8)	
Self employed	91	59 (64.8)	
Formerly employed	78	40 (51.3)	
**Distance to nearest facility (km)**			5.35, **0.021**
> 5	85	61 (71.8)	
≤ 5	255	147 (57.6)	
**No. of ANC visits**			6.47, **0.011**
< 4 visits	199	133 (68.8)	
≥ 4 visits	141	75 (53.2)	
**Perceived availability of HMs**			1.51, 0.219
Easily available	57	39 (68.4)	
Not easily available	283	169 (59.7)	
**Perceived safety of HMs**			11.74, **<0.01**
Safe	83	64 (77.1)	
Not safe	257	144 (56.0)	
**Health care provider’s stance towards use of HMs**			13.15**, < 0.01**
Did not discourage	131	96 (73.3)	
Discouraged use	209	112 (53.6)	

^*^Pearson’s chi-square

^**a**^ 12 missing values

In Table 2, we present independent factors associated with HM use during the most recent pregnancy or delivery. Significant predictors of HMs use were number of ANC visits and maternity health care provider’s advice against the use of HMs. The adjusted prevalence ratio of using HMs was 24 % higher among women who made less than four visits to the ANC as compared to women who made four or more visits (aPR: 1.24; 95%CI: 1.02, 1.50, *p* = 0.03). Furthermore, the aPR of using HMs was 35 % higher among women who were not discouraged by the maternity health care providers against using HMs during pregnancy or delivery versus those who were discouraged (aPR: 1.35; 95%CI: 1.13, 1.60, *p* = 0.01)

**Table 2:** Poisson regression analysis of predictors of herbal medicine use during pregnancy or delivery in Tabora, Tanzania (*N* = 340)

Factor	HM use Number (%)	Prevalence Ratio (95 % CI)		
		**Crude**	**Adjusted**	***p*-value**
**Occupation of mother**				
Peasant/housewife	108 (61.0)	0.81 (0.65, 1.02)	0.83 (0.64, 1.07	0.14
Self-employed	73 (57.5)	0.77 (0.60, 0.98)	0.89 (0.69, 1.17)	0.42
Formerly employed	27 (75.0)	Reference	Reference	
**Occupation of spouse**				
Peasant	103 (64.8)	1.26 (0.99, 1.61)	1.17 (0.90, 1.51)	0.24
Self employed	59 (64.8)	1.26 (0.97, 1.65	1.15 (0.88, 1.49)	0.32
Formerly employed	40 (51.3)	Reference	Reference	
**No. of ANC visits**				
< 4 visits	133 (68.8)	1.26 (1.05,1.51)	1.24 (1.02, 1.50)	**0.03**
≥ 4 visits	75 (53.2)	Reference	Reference	
**Distance to nearest health facility (km)**				
> 5	61 (71.4)	1.25 (1.05,1.48)	1.09 (0.90, 1.31)	0.37
≤ 5	147 (57.6)	Reference	Reference	
**Perceived safety of HMs**				
Safe	64 (77.1)	1.38 (1.17, 1.61)	1.12 (0.87, 1.42)	0.40
Not safe	144 (56.0)	Reference	Reference	
**Health care provider’s stance towards HMs use**				
Did not discourage	96 (73.3)	1.37 (1.16, 1.61)	1.35 (1.13, 1.60	**0.01**
Discouraged use of HMs	112 (53.6)	Reference	Reference	

## Discussion

This study determined the magnitude of HMs use and associated factors during pregnancy or delivery among women who delivered a live-born baby between September 2016 and September 2018, in Tabora, Tanzania. Findings show that use of HMs is high, (60 %) and the independent predictors of use include frequency of ANC visits and the stance of maternity health care providers.

Worldwide, use of HMs has grown considerably among pregnant women, and particularly in sub-Saharan Africa [5]. Similar to the findings from this study, the level of use of HMs is high in other areas of sub-Saharan Africa, Asia and Middle East, where the proportions of use range between 20 and 80 % [9, 11, 22–24]. For many years, women have used HMs for remedial of several conditions during pregnancy and in the delivery process. Findings from our study are consistent with reports from other countries that pregnant women use herbs for different purposes, including easing labour pains, accelerating labour, increasing milk production, and aiding postpartum uterine involution [13, 22, 25]. The grounds for the high usage of HMs could be attributed to their easy accessibility and the general lack of awareness of their potential side effects [26–28].

Women who perceive HMs as safe during pregnancy or delivery tend to use them more than those who perceive them as unsafe [10, 24, 29]. In the current study, the prevalence ratios of using HMs were between 10 and 40 % higher among women who perceived HMs as safe versus those who perceived them as unsafe. Despite lack of significant association between perceived safety and use of HMs during pregnancy, the assertion women have about safety of these herbs will only make scientific sense when they are authentically tested, standardized, and quality controlled [30]. Thus, the high rates of HMs usage in relation to pregnancy should be of concern. It is even more serious as the unproven perceived safety may lead to rapid increase in promotion of HMs in the society as well as the media. It is common to see posters advertising such medicines and herbalists in various parts of Tanzania. In such advertisements, HMs are often promoted as natural and safe, attracting their wide use, especially among pregnant women who are naturally concerned about the whole process of childbirth and health of the unborn child.

Plants used for HMs are less expensive as compared to modern medicines and have been culturally considered as effective and an acceptable option even when modern health facilities are available [31]. Notwithstanding their easy availability and perceived effectiveness, many countries with high usage also have generally poor quality of health services and lower hygienic standards than countries with lower usage. Furthermore, some of these countries with high usage of HMs are also reporting high maternal and newborn morbidity and mortality, suggesting a connection between use of HMs and adverse pregnancy outcomes. In rural Malawi for instance, where 25.7 % of pregnant women used a popular herb, *mwanamphepo*, the odds of maternal morbidity were 28 % higher among self-reported users than non-users of *mwanamphepo*. Furthermore, the probabilities of neonatal morbidity or death were 22 % higher among neonates whose mothers reported use of *mwanamphepo* than those who did not [28]. Significantly higher odds of having postnatal complications have, likewise, been observed among women in Tanzania who reported use of local herbs during pregnancy or delivery versus those who did not [17]. A report from Northern Italy also suggests an increased risk of giving birth to preterm babies among women who regularly rub almond oil on the pregnant abdomen compared with non-users [18]. Although we cannot completely rule out the effect of pressure exerted on a pregnant abdomen through rubbing with herbs, the safety of any medicine, including HMs, cannot be guaranteed in pregnancy because of the possible teratogenic effects [32].

Physical accessibility to health care facilities is an important attribute to using HMs during pregnancy [6, 16, 24, 27, 33]. In the current study, women considered to be living far from the nearest health facility had an increased likelihood of using HMs compared to women living closer to health facilities. However, the association between use of HMs and distance to facility in this study was not statistically significant. This is most probably due to the fact that respondents were from an urban setting where health care facilities are concentrated. Despite this, long distances may contribute to women delivering under the care of unskilled attendants and hence exposure to the use of medicinal herbs. However, there might also be secret exposures even when a birth takes place in a health care facility. For instance, a study among healthcare professionals in Scotland revealed that a third of the respondents, significantly more midwives recommended use of complementary and alternative therapies to pregnant women [34].

Whereas response to high exposures to HMs during pregnancy or delivery is essential, the most important concern is lack of awareness and knowledge among pregnant women [15] and the community in general about their potential side effects on the mother and the fetus [6, 35]. Findings from this study are similar to observations from other countries where self-medication with HMs during pregnancy or delivery is common, but very few women attending ANC services receive information on their adverse effects. In Kenya for instance, merely 14 % of pregnant women received health advice from healthcare workers [36]. In our study, women who were advised by the maternity health care providers against the use of HMs reported lower usage compared to their counterparts. This suggests that provision of appropriate information regarding use of HMs during pregnancy may curtail their injudicious use. In Tabora, all respondents received ANC services at least once during the most recent birth. Therefore, opportunities for healthcare workers to discuss the use of HMs during pregnancy are available.

Data in the current study were collected in RMCH clinics among mothers bringing their under-five children for vaccinations and growth monitoring. According to Tanzania Demographic and Health Surveys, the percentage of children who do not receive any vaccination has been declining, from 4 % in 2004-05 to 2 % in 2010 and 2015-16. This suggests that RMCH clinics are a viable capture point for most mothers that have recently delivered. Additionally, service provision in RMCH clinics usually involves discussions and dialogue with health care providers. Normalization of discussions in this setting may explain the observed high rates of participation in interviews in this study. Similar high participation rates have likewise been documented in other areas in Tanzania where almost 98 % of women with children up to two years completed a questionnaire [17]. Although we are not explicitly positive in assessing the causal relationship due to the nature of the study design, in our case, the main circumstance embracing external validity may include the large sample size. However, the external validity of the results would also depend on time changes following the extent of interventions instituted as this was an observational and not an experimental study.

This study has several potential limitations that readers must consider when interpreting the findings. First, although we assumed that women who gave birth within the preceding two years were likely to remember use of HMs in their most recent pregnancy or delivery; we are unable to completely rule out the possibility of recall bias. If some women were unable to remember, this bias could have contributed to the low estimates. Second, the study recruited only women who had a live-born baby, which might also have underestimated the outcome variable. Third, some of the women were aware that use of HMs during pregnancy was discouraged by health care providers. Therefore, some respondents might have provided socially desirable responses, concealing reporting use of HMs and thus under-estimating the proportion. Despite all these, to the best of our knowledge, this is the first study attempting to assess use of HMs during pregnancy or delivery as a forgotten exposure when adopting strategies to attain the SDG 3 target on reduction of maternal and newborn morbidity and mortality.

## Conclusions

The rate of herbal medicine use among pregnant women in Tanzania is high, 60 %. Significant predictors of use include frequency of attending ANC and health care providers’ stance towards the use of HMs. Maternity health care providers are crucial for promotion of proven and safe interventions during pregnancy and delivery if pregnant women make the recommended number of antenatal visits. Further research is required to understand the magnitude, patterns and predictors of HM use among women during pregnancy or delivery, in both urban and rural settings as a fundamental step towards achieving SDG 3.

## Supplementary Information

**Additional file 1.**

## Data Availability

All data generated or analyzed during this study are included in this article and are available upon request from the corresponding author.

## Abbreviations

ANC: Antenatal clinic
aPR: :Adjusted prevalence ratio
CI: Confidence interval
HMs: Herbal medicines
RMCH: Reproductive, maternal and child health
SDG: Sustainable Development Goal
