# Supplementary material for: Understanding forgotten exposures towards achieving Sustainable Development Goal 3: a cross‐sectional study on herbal medicine use during pregnancy or delivery in Tanzania
Source: BMC Pregnancy Childbirth. 2021 Apr 1;21:270. doi: 10.1186/s12884-021-03741-5 (PMC8017693; doi:10.1186/s12884-021-03741-5)
Supplement: Supplementary file 1 — Additional file 1. [file 12884_2021_3741_MOESM1_ESM.docx]

**English Questionnaire on the use of herbal medicines during pregnancy or delivery among women in Tabora Municipality.**

Hello Madam, good morning/afternoon.

My name is ……….. I am here on behalf of the principal investigator from the School of Public Health, Muhimbili University of Health and Allied Sciences. We are doing a research on the use of herbal medicines among women who delivered a live born baby within the past two years in Tabora municipality. The aim of this study is to asses use of herbal medicines during pregnancy or delivery and to find out factors associated with the practice. I am going to ask you a few questions and please respond sincerely. All the information you provide shall be treated confidentially and will not be disclosed to anybody. Your participation in this study is voluntary, and please not that you are free to decline answering any question which you do not feel comfortable to respond to. I hope you have understood me so please let us begin.

| **No** | | **Question** | **Response** |
| --- | --- | --- | --- |
|  | | Date of interview: | ☐☐/☐☐/2018  (dd/mm/yyyy) |
|  | | Questionnaire number: | ……………… |
| 1 | | What is your age in years? | Age (complete yrs)………. |
| 2 | | How many times have you delivered a live born baby? | 1.One  2.Two  3.Three or more |
| 3 | | What is your current marital status? | 1. Single  2. Married  3. Divorced/separated  4. Widow |
| 4 | | What is your main occupation? | 1. Peasant  2. Housewife  3. Self employed  4. Formerly employed |
| 5 | | What is your partner’s occupation? | 1. Peasant  2. Self employed  3. Formerly employed |
| 6 | | What is your highest level of education? | 1. No formal education  2. Incomplete primary school  3. Completed primary school  4. Secondary education  5. University/College education |
| 7 | Are herbal medicines easily available as compared to conventional medicines | 1. Yes  2. No |  |
| 8 | Have you ever used herbal medicines during pregnancy? | 1. Yes  2. No |  |
| 9 | In the last pregnancy at what gestational age did you start antenatal services? | 1. I don’t remember  2. Less than three months  3. Three or more months |  |
| 10 | How many times did you attend antenatal clinic during previous pregnancy? | 1. None  2. Less than four times  3. Four or more times |  |
| 11 | Were you satisfied with the services provided at antenatal clinic? | 1. Yes  2. No |  |
| 12 | If NO, Why were you not satisfied with the antenatal clinic services? | 1. Bad language from health care providers  2. Distance  3. High cost  4. Others (Mention) |  |
| 13 | Are you paying for the services provided at antenatal clinic? | 1. Yes  2. No |  |
| 14 | How far is the nearest public health facility with antenatal services? | 1. Less than 5km  2. 5 km to 10 km  3. More than 10 km |  |
| 15 | Did you use herbal medicines during the past pregnancy or delivery? | 1. Yes  2. No |  |
| 16 | In how many occasions did you use herbal medicines during the last pregnancy? | 1. Once  2. Twice  3. More than three times |  |
| 17 | Through which route did you mostly use the herbal medicines? | 1. Oral  2. Inserted into the vagina  3. Rubbed on the abdomen |  |
| 18 | Please, mention any complications which may occur following use of herbal medicines in pregnancy | 1. None  2. Death of mother and or foetus  3. Infection  4. Uterus rupture  5. Excessive bleeding  6. Exhaustion of foetus/mother |  |
| 19 | Who mostly advised you to use herbal medicines during that pregnancy? | 1. Nobody/self  2. Husband/spouse  3. Parents  4. Traditional birth attendant  5. Friend |  |
| 20 | What was the main reason for using herbal medicines during labour? | 1. Relieve labour pain  2. Shorten duration of labour  3. Prevent post-delivery bleeding  4. Increase milk secretion |  |
| 21 | In what form/preparation are herbal medicines available in your locality | 1. Powder  2. Solution  3. Roots/tubers  4. Leaves |  |
| 22 | At what time are herbal medicines used for labour purposes? | 1. Before onset of labour  2. During labour  3. During delivery |  |
| 23 | Whom do you usually consult for pregnancy related advice while at home? | 1. Nobody  2. Husband/spouse  3. Mother in law  4. Friends  5. Others (specify………….....) |  |
| 24 | Are herbal medicines safe to pregnant women? | 1. Yes  2. No |  |
| 25 | During the last pregnancy were you discouraged by a health provider at the clinic against the use of herbal medicines? | 1. Yes  2. No |  |

THIS IS THE END OF OUR INTERVIEW AND THANK YOU FOR YOUR TIME
